# Intravitreal rezafungin and anidulafungin in a rabbit model of *Aspergillus fumigatus* endophthalmitis

**DOI:** 10.1038/s41598-026-53347-0

**Published:** 2026-05-27

**Authors:** Yue Zhang, Yanjie Zhou, Yi Tang, Hong Wu

**Affiliations:** 1 Department of Ophthalmology, The Second Hospital of Jilin University, 218 Ziqiang Street, Nanguan District, Changchun, 130000 Jilin China; 2 Department of Ophthalmology, Changzhi People’s Hospital, Changzhi, Shanxi China

**Keywords:** Fungal endophthalmitis, *Aspergillus fumigatus*, Rezafungin, Anidulafungin, Liposomal amphotericin B, Voriconazole, Diseases, Drug discovery, Medical research, Microbiology

## Abstract

Rezafungin (RZF) and anidulafungin (AFG) are echinocandins with potential for treating *Aspergillus fumigatus* endophthalmitis. We evaluated their antifungal and anti-inflammatory effects in vitro and in a rabbit endophthalmitis model. In vitro, minimum effective concentrations and minimum inhibitory concentrations were determined, biofilm inhibition was assessed, and drug-induced structural damage was examined by confocal laser scanning microscopy, scanning electron microscopy and transmission electron microscopy. TNF-α and IL-1β mRNA levels in RAW 264.7 macrophages were measured to assess inflammatory regulation. In vivo, a rabbit model of *A. fumigatus* endophthalmitis was established and, 24 h after infection, eyes received a single intravitreal injection of liposomal amphotericin B, voriconazole, RZF, AFG or normal saline. Clinical inflammation scores, intraocular fungal burden, aqueous humor TNF-α levels and histopathological changes were compared among groups. RZF and AFG showed low minimum effective concentrations, inhibited biofilm formation, disrupted fungal cell wall and membrane integrity in vitro and significantly reduced intraocular inflammation, fungal burden and aqueous humor TNF-α in vivo, with overall efficacy comparable to that of liposomal amphotericin B and voriconazole. These findings support RZF and AFG as promising alternative agents for intravitreal therapy of *A. fumigatus* endophthalmitis.

## Introduction

Fungal endophthalmitis is an uncommon but highly destructive intraocular infection that, if not treated promptly, can cause irreversible retinal damage and permanent vision loss^1^. Among cases of exogenous endophthalmitis, filamentous fungi such as *Aspergillus fumigatus* (*A. fumigatus*) are major pathogens, particularly following ocular trauma or postoperative infection. The disease progresses rapidly, responses to current antifungal therapies are often inconsistent, and visual outcomes are generally poor^2^. Current first-line management typically consists of pars plana vitrectomy combined with intravitreal injection of amphotericin B or voriconazole (VCZ), along with systemic antifungal therapy^3^. However, delayed microbiological diagnosis, drug-related toxicities, and unfavorable pharmacokinetics limit treatment efficacy, so that even aggressive combination therapy often fails to achieve satisfactory visual outcomes^4^. One key factor is the poor penetration of antifungal agents across the blood–ocular barrier, which makes it difficult to achieve effective drug concentrations in the vitreous cavity^5^.

Echinocandin antifungals act by non-competitively inhibiting fungal 1,3-β-d-glucan synthase, thereby blocking cell-wall β-glucan synthesis and disrupting the integrity of the fungal cell wall^6,7^. This class includes caspofungin, micafungin, and anidulafungin (AFG), which exhibit rapid fungicidal activity against *Candida* species but are mainly fungistatic against *Aspergillus* species^6^. Accordingly, echinocandins are usually reserved as second-line or salvage therapy for invasive aspergillosis, sometimes in combination with other antifungal agents^8^. Because echinocandins have a large molecular weight and high protein binding, systemic administration results in limited penetration into the vitreous, making it difficult to achieve effective intraocular concentrations at the site of infection^9^. Nevertheless, animal studies suggest that intravitreal administration is feasible: in a rabbit model of *Candida* endophthalmitis, a single intravitreal injection of 50 µg anidulafungin achieved efficacy comparable to voriconazole and amphotericin B without obvious ocular toxicity^10^. These findings indicate that intravitreal echinocandins may represent an effective alternative treatment for intraocular fungal infections.

Rezafungin (RZF) is a next-generation echinocandin derived from anidulafungin, and in 2023 it was approved by the U.S. Food and Drug Administration for the treatment of invasive candidiasis in adults^11^. RZF has a markedly prolonged half-life of approximately 130 h, allowing for once-weekly dosing^12,13^. In vitro studies have shown that RZF exhibits broad and potent inhibitory activity against multiple fungal species, including low MECs against *A. fumigatus* (MEC₅₀/₉₀ of approximately 0.015/0.03 µg/mL), and retains good activity against some azole-resistant clinical isolates^14,15^. Recent animal experiments further demonstrated that systemic RZF significantly reduces intraocular fungal burden and inflammation in a rabbit model of *Candida* endophthalmitis, with efficacy superior to micafungin and voriconazole^9^. However, experimental data on the safety and efficacy of intravitreal RZF for *A. fumigatus* endophthalmitis are still lacking.

Against this background, the present study aimed to systematically evaluate the antifungal and anti-inflammatory efficacy of a single intravitreal injection of rezafungin and anidulafungin in a rabbit model of *A. fumigatus* endophthalmitis, with the goal of providing a new therapeutic option for clinical practice.

## Results

### Rezafungin and anidulafungin exhibit low MECs against *A. fumigatus*

The MECs of RZF and AFG against *A. fumigatus* ranged from 0.0156 to 0.0313 µg/mL. The MICs of liposomal amphotericin B (L-AmB) and VCZ against *A. fumigatus* were both 0.25–0.5 µg/mL.

### Echinocandins can effectively inhibit the formation of *A. fumigatus* biofilms

**Fig. 1:**
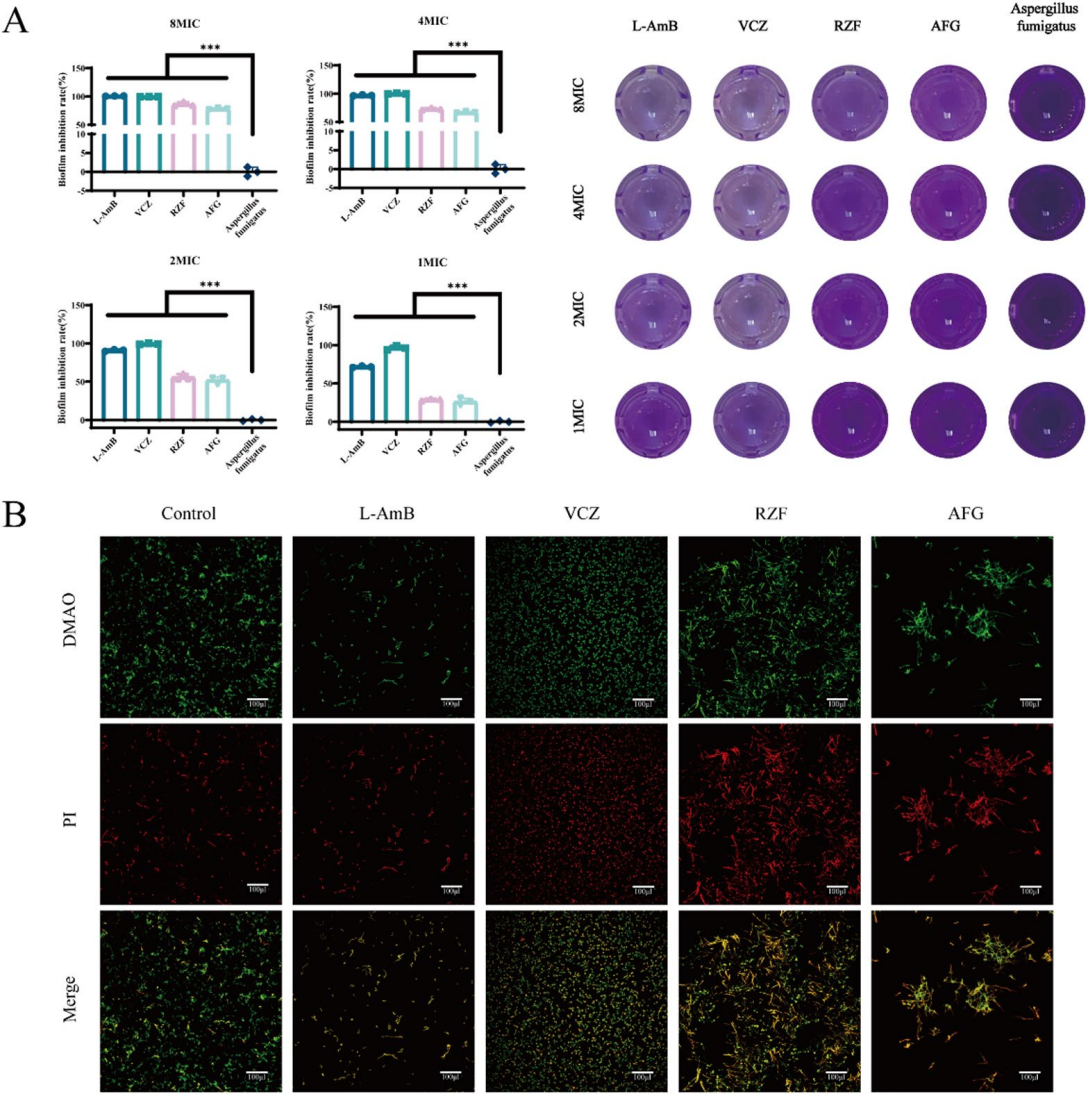
(**A**) Inhibition rates of L-AmB, VCZ, RZF, and AFG on *A. fumigatus* biofilm formation at different concentrations as determined by crystal violet staining. (**B**) CLSM images of *A. fumigatus* biofilms co-stained with DMAO and PI after treatment with the four drugs at 4× MIC (Scale bar: 100 μm).

All four drugs (L-AmB, VCZ, RZF, AFG) at 1×, 2×, 4×, or 8× MIC significantly inhibited the formation of *A. fumigatus* biofilms, and the inhibitory effect was concentration-dependent (Fig. 1A). At 8× MIC, the inhibition rates of L-AmB, VCZ, RZF, and AFG on biofilm formation were 100%, 100%, 86.16%, and 78.17%, respectively; at 4× MIC, the inhibition rates were 97.20%, 100%, 73.14%, and 67.09%, respectively; at 2× MIC, the inhibition rates were 91.05%, 99.47%, 56.25%, and 52.12%, respectively; at 1× MIC, the inhibition rates were 71.78%, 96.43%, 28.89%, and 26.29%, respectively. These results confirm that both echinocandins (RZF, AFG) and traditional antifungals (L-AmB, VCZ) can significantly inhibit the formation of *A. fumigatus* biofilms at different concentrations.

The membrane integrity of *A. fumigatus* treated with the four drugs was further evaluated using DMAO and propidium iodide (PI) staining followed by confocal laser scanning microscope (CLSM) (Fig. 1B). In the control group, only minimal PI staining was observed. In contrast, treatment with 4× MIC of any drug significantly increased PI fluorescence, indicating substantial membrane damage.

### Echinocandins mediate fungicidal effects by disrupting the integrity of fungal cell walls and membranes

**Fig. 2:**
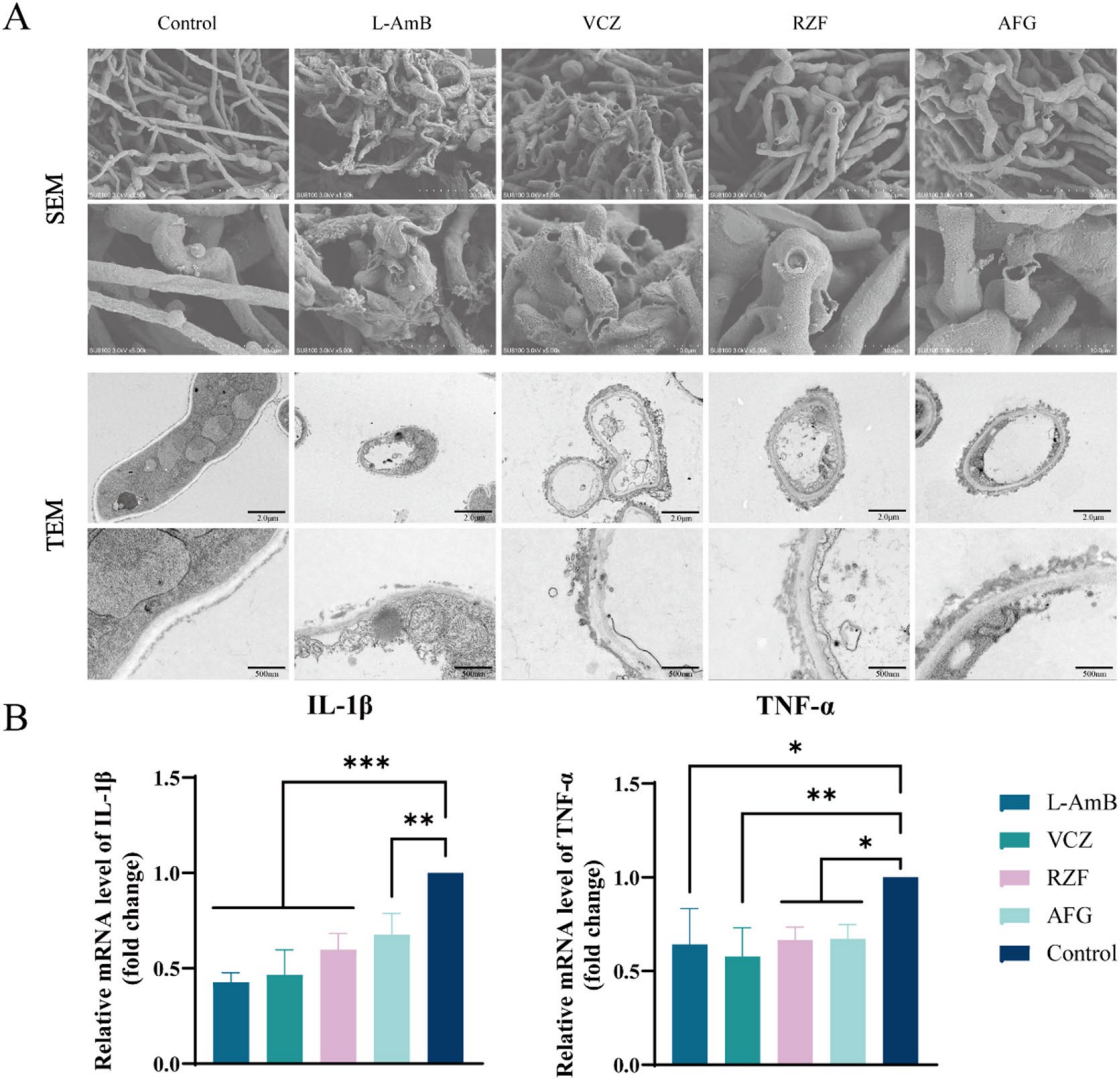
(**A**) SEM and TEM images of *A. fumigatus* after treatment with the four drugs at 4× MIC for 48 h (SEM scale bars: 30 μm/10 µm; TEM scale bars: 2 μm/500 nm). (**B**) mRNA expression levels of TNF-α and IL-1β after co-culture of *A. fumigatus* and RAW 264.7 cells treated with the four drugs at 40× MIC for 4 h.

Scanning electron microscope (SEM) revealed that untreated *A. fumigatus* exhibited an elongated morphology with intact cell walls. In contrast, all four antifungal drugs (L-AmB, VCZ, RZF, AFG) induced significant morphological abnormalities, including cellular shrinkage, rupture, and collapse, accompanied by blurred cell boundaries. Transmission electron microscope (TEM) further confirmed intact ultrastructure in controls, whereas drug-treated cells showed disintegration of cell walls/membranes, disorganized cytoplasmic electron density, and organelle destruction (Fig. 2A).

### Echinocandins effectively downregulate inflammatory factors expression in vitro

qRT-PCR analysis showed that after RAW 264.7 cells were co-cultured with *A. fumigatus* and drugs at 40× MIC for 4 h, the mRNA levels of TNF-α and IL-1β were significantly reduced compared to the untreated control (Fig. 2B).

### Rabbit model of *A. fumigatus* endophthalmitis

**Fig. 3:**
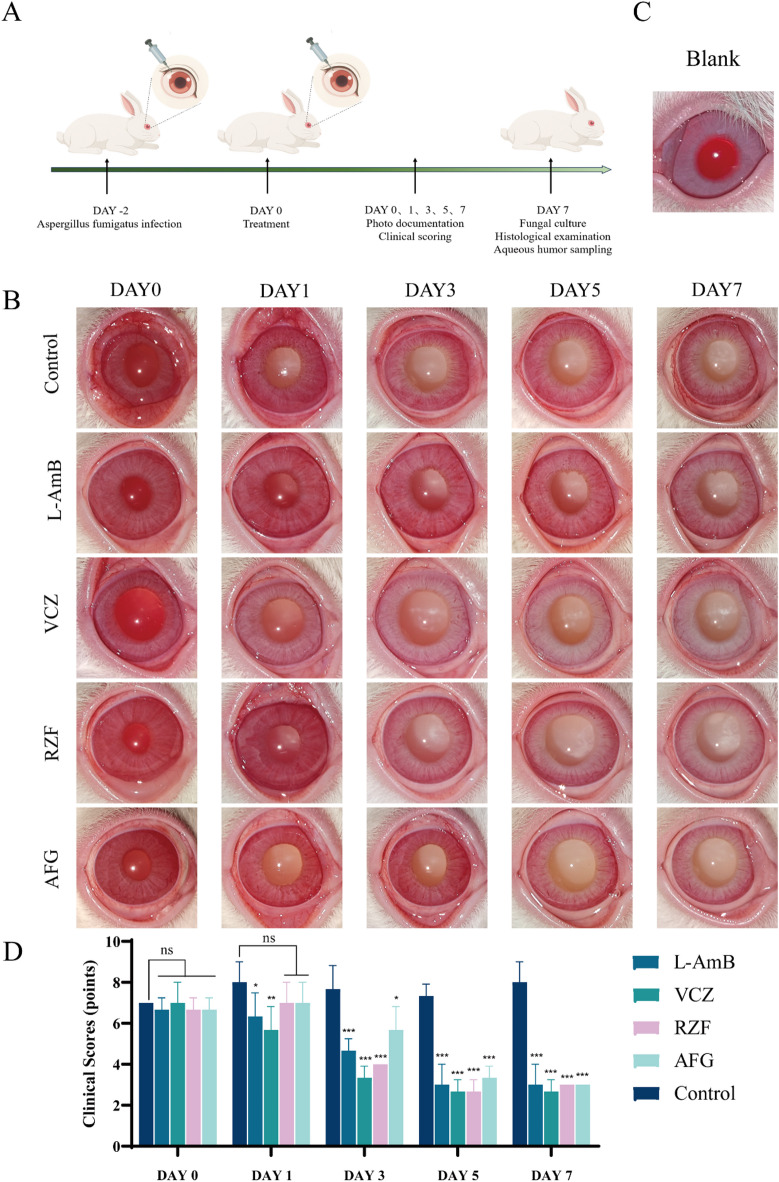
(**A**) Timeline for modeling, therapeutic intervention, and evaluation of *A. fumigatus* endophthalmitis infection. (**B**) Therapeutic effects of intravitreal injection of normal saline, L-AmB, VCZ, RZF, and AFG in rabbit eyes for treating fungal endophthalmitis at Day 0, Day 1, Day 3, Day 5, and Day 7. (**C**) Representative external photograph of a healthy, uninfected rabbit eye. (**D**) Clinical scoring results of each group at Day 0, Day 1, Day 3, Day 5, and Day 7 after drug injection.

The experimental timeline is shown in Fig. 3A. At 24 h post-infection (Day 0), all eyes exhibited typical endophthalmitis, including corneal edema, mild conjunctival hyperemia and edema, pronounced iris hyperemia, and mild vitreous opacity. Control eyes worsened over time, showing localized corneal edema, conjunctival exudation, and iris hyperemia, as well as complete vitreous opacity with loss of the red reflex by Day 7. All four drug treatments effectively halted disease progression. VCZ performed better than the other three groups in corneal repair speed and iris inflammation control. Mild iris hyperemia persisted in the AFG group on Day 7, suggesting a potentially delayed anti-inflammatory effect. However, all treatment groups eventually showed comparable outcomes by the end of the study (Fig. 3B). A representative external photograph of a healthy, uninfected rabbit eye imaged under the same conditions is provided as a normal reference (Fig. 3C).

Clinical scores indicated no significant differences among any groups on Day 0 (*P* > 0.05). On Day 1, only the L-AmB and VCZ groups differed significantly from the control group (*P* < 0.05), and no significant differences were observed between the RZF/AFG groups and the control group (*P* > 0.05). On Day 7, no significant differences were observed between the RZF/AFG groups and the traditional drug groups (L-AmB/VCZ) (*P* > 0.05), whereas all four treatment groups differed significantly from the control group (*P* < 0.05) (Table 1; Fig. 3D).

**Table 1:** Clinical scoring results of endophthalmitis in each group.

Groups	Time points				
	DAY 0	DAY 1	DAY 3	DAY 5	DAY 7
Control	7.00 ± 0.00	8.00 ± 1.00	7.67 ± 1.15	7.33 ± 0.58	8.00 ± 1.00
L-AmB	6.67 ± 0.58	6.33 ± 1.15	4.67 ± 0.58	3.00 ± 1.00	3.00 ± 1.00
VCZ	7.00 ± 1.00	5.67 ± 1.15	3.33 ± 0.58	2.67 ± 0.58	2.67 ± 0.58
RZF	6.67 ± 0.58	7.00 ± 1.00	4.00 ± 0.00	2.67 ± 0.58	3.00 ± 0.00
AFG	6.67 ± 0.58	7.00 ± 1.00	5.67 ± 1.15	3.33 ± 0.58	3.00 ± 0.00

**Fig. 4:**
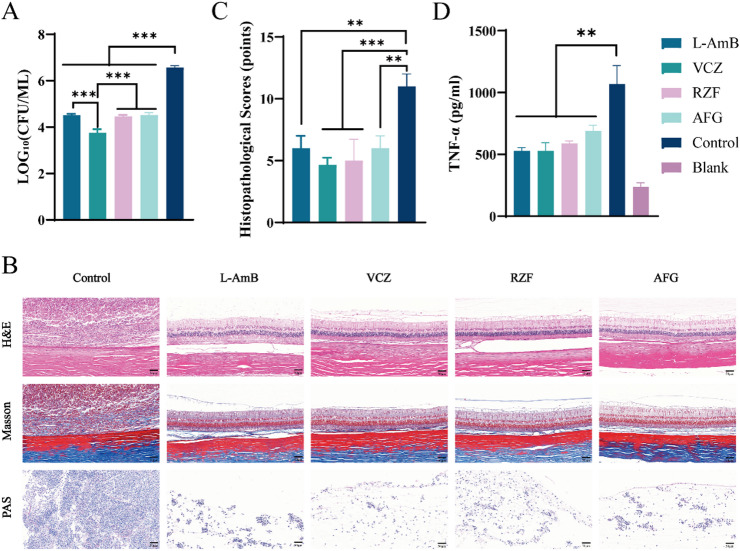
(**A**) Results of dilution plating counting of eyeballs in each group at Day 7. (**B**) Results of retinal H&E, Masson and PAS staining (scale bars: 50 μm) in each group at Day 7. (**C)** Pathological section scoring results of each group at Day 7. (**D**) TNF-α expression levels in aqueous humor of each group at Day 7 as determined by ELISA.

Fungal burden analysis showed significantly reduced colony counts in all treatment groups compared to the control (Fig. 4A). All four treatment groups achieved fungicidal rates exceeding 99% (Table 2).

**Table 2:** Results of dilution plating counting and fungicidal rates in different groups.

Groups	LOG10 (CFUs/mL)	Fungicidal rates (%)
Control	6.58 ± 0.07	0
L-AmB	4.52 ± 0.05	99.131 ± 0.103
VCZ	3.76 ± 0.15	99.844 ± 0.049
RZF	4.46 ± 0.06	99.233 ± 0.103
AFG	4.52 ± 0.10	99.113 ± 0.186
*P*	< 0.05	< 0.05

Pathological analysis revealed that in the control group, diffuse inflammatory cell infiltration, fibrinous exudation, and necrotic debris were observed in the vitreous cavity, and the retina presented full-thickness necrosis. In contrast, all treatment groups (L-AmB, VCZ, RZF, AFG) showed focal inflammatory cells scattered in the vitreous cavity, with the retinal tissue structure roughly intact albeit with minor morphological changes. Periodic acid–Schiff (PAS) staining revealed that a large number of dense purple-red *A. fumigatus* clumps were present in the vitreous cavity of the control group, while all drug groups exhibited markedly reduced fungal loads (Fig. 4B). Pathological scores were significantly lower in all treatment groups compared to the control, with no statistical difference between echinocandins (RZF, AFG) and the traditional drugs (L-AmB, VCZ) (Table 3; Fig. 4C).

**Table 3:** Histopathological scores.

Groups	Histopathological scores
Control	11.00 ± 1.00
L-AmB	6.00 ± 1.00
VCZ	4.67 ± 0.58
RZF	5.00 ± 1.73
AFG	6.00 ± 1.00

ELISA quantification of TNF-α in rabbit aqueous humor showed that all drug-treated groups exhibited significantly lower TNF-α levels compared to the control (*P* < 0.001) (Fig. 4D).

## Discussion

This study provides the first experimental evidence that a single intravitreal injection of the next-generation echinocandin RZF or AFG produces significant therapeutic effects in a rabbit model of *A. fumigatus* endophthalmitis, with antifungal and anti-inflammatory efficacy comparable to L-AmB and voriconazole (VCZ). Our in vitro data showed MECs of 0.0156–0.0313 µg/mL for RZF and AFG against *A. fumigatus*, indicating strong inhibitory activity. These findings are consistent with previous reports of RZF MEC₅₀/₉₀ values of approximately 0.015/0.03 µg/mL for clinical *A. fumigatus* isolates^15,16^ and with the documented in vitro activity of AFG in invasive aspergillosis^10^. Compared with the MICs of L-AmB and VCZ (0.25–0.5 µg/mL), RZF and AFG exhibited greater in vitro potency, in line with the superior MEC profile of RZF reported in multicenter surveillance studies of *Aspergillus* isolates^17^. Consistent with the notion that biofilms are a key mechanism underlying the recalcitrant nature of endophthalmitis^18^, we found that all four drugs significantly inhibited *A. fumigatus* biofilm formation at various multiples of the MIC. However, at the same multiple of the MIC, echinocandins (4× MIC inhibition rate 67%–73%) were less effective than L-AmB and VCZ (> 97%), which may reflect their primary action on cell-wall β-glucan synthesis rather than direct disruption of the mature biofilm matrix, whereas L-AmB and VCZ can more efficiently dismantle biofilms by damaging membranes or interfering with ergosterol synthesis^19^. Electron microscopy and live/dead staining showed that all four agents induced abnormal hyphal morphology, disruption of cell-wall and cell-membrane structures, and disorganization of intracellular contents. These findings suggest that although echinocandins may be slightly less effective than traditional agents at clearing mature biofilms, they still exert marked inhibitory effects during early adhesion and hyphal growth, providing a mechanism complementary to drugs that target ergosterol.

With respect to inflammatory regulation, RZF and AFG significantly reduced TNF-α and IL-1β mRNA levels in *A. fumigatus*-stimulated RAW 264.7 cells, suggesting that beyond lowering pathogen burden they may also protect the host by directly or indirectly modulating inflammatory responses. This observation is in line with recent reports that systemic RZF markedly attenuates inflammatory damage in a rabbit model of *Candida* endophthalmitis, further supporting its anti-inflammatory potential^9^. Importantly, in the rabbit *A. fumigatus* endophthalmitis model, a single intravitreal injection of RZF or AFG yielded clinical inflammation scores, fungal burden, histological damage, and aqueous TNF-α levels that were comparable to those seen with L-AmB and VCZ, and all treatment groups achieved fungicidal rates above 99%. Although VCZ showed a slight advantage in early control of inflammation and corneal healing, and iris hyperemia resolved more slowly in the AFG group, all treatment groups ultimately achieved good inflammatory control over time, indicating that echinocandins are not inferior to traditional drugs in overall efficacy.

As a next-generation echinocandin, RZF offers pharmacokinetic advantages such as a prolonged half-life and high chemical stability, enabling once-weekly systemic dosing while maintaining adequate drug exposure^8,20^. Although intraocular pharmacokinetics were not assessed in this study, the comparable efficacy achieved with a single intravitreal injection of RZF relative to L-AmB and VCZ suggests that RZF may also have prolonged tissue retention in the eye, potentially reducing the need for repeated injections—an advantage of practical importance in fungal endophthalmitis requiring long-term follow-up and multiple interventions. Moreover, previous in vitro studies have shown that RZF maintains good activity against multiple azole-resistant *A. fumigatus* isolates, offering a potential new option for patients with azole-resistant infections or azole intolerance^21^.

Naturally, this study has several limitations. First, we used only a single reference *A. fumigatus* strain and did not include multiple clinical isolates or azole-resistant strains, which may underestimate inter-strain variability. Second, we evaluated only a single intravitreal dose of 35 µg, without comparing different dose levels or repeated-dosing regimens, so the optimal dosing strategy remains unclear. Third, the observation period was limited to 7 days, which did not allow assessment of long-term retinal structural and functional recovery. Fourth, we did not perform intraocular pharmacokinetic measurements, preventing a direct correlation between vitreous drug concentrations, efficacy, and potential toxicity. Fifth, in vitro cytokine transcription was assessed only in RAW 264.7 macrophages, and ARPE-19 models were not included. Sixth, immunohistochemical staining for inflammatory markers and quantitative inflammatory cell infiltration analysis were not performed. Finally, in vitro susceptibility testing was conducted in PDB rather than CLSI-recommended RPMI 1640, which may affect absolute MEC/MIC values and limits direct comparison with CLSI-based datasets. These issues need to be addressed in future studies.

In conclusion, this study demonstrates that a single intravitreal injection of RZF or AFG provides potent antifungal and marked anti-inflammatory effects in *A. fumigatus* endophthalmitis, with efficacy comparable to current standard therapies, thereby providing important experimental support for the use of echinocandins in refractory filamentous fungal endophthalmitis.

## Materials and methods

### *A. fumigatus* culture

Potato dextrose agar (PDA; Qingdao Hope Bio-Technology Co., Ltd.) was prepared by dissolving 47 g of medium in 1000 mL of distilled water, autoclaving at 121 °C for 15 min, and pouring the solution into 90-mm Petri dishes to solidify. Potato dextrose broth (PDB; Beijing Solarbio Science & Technology Co., Ltd.) was prepared by dissolving 35 g of medium in 1000 mL of distilled water and autoclaving at 121 °C for 15 min. Blocks of the standard *A. fumigatus* strain (BNCC 272175) were aseptically inoculated onto the center of PDA plates and incubated at 30 °C for 5–7 days. After incubation, conidia were harvested by gently scraping the surface of the plate and suspended in sterile saline containing 0.01% (v/v) Tween 20. The spore suspension was filtered and adjusted to the desired concentration (0.4 × 10⁴ to 5 × 10⁴ CFU/mL) using a hemocytometer.

### In vitro susceptibility testing

Antifungal susceptibility testing was performed by a broth microdilution assay modified from CLSI M38 (3rd ed.)^22^. PDB was used as the test medium instead of the CLSI-recommended RPMI 1640. For RZF (Abmole) and AFG (Abmole), the MEC was defined as the lowest concentration that produced characteristic morphological changes (short, thick, abnormally branched hyphae) after 24 h of incubation at 30 °C. For L-AmB (Ambisome) and VCZ (Livzon), the MIC was defined as the lowest concentration that resulted in 100% growth inhibition after 48 h of incubation. The concentration range tested for RZF and AFG was 0.0078–4 mg/L, and for L-AmB and VCZ it was 0.03–16 mg/L.

### Biofilm inhibition assay

Biofilm formation by *A. fumigatus* was assessed in 96-well plates using crystal violet staining. Briefly, 100 µL of an *A. fumigatus* suspension (1 × 10^4^ CFU/mL) was added to each well, followed by 100 µL of drug solution at 1×, 2×, 4×, or 8× MIC. After 48 h of incubation at 30 °C, the culture was gently removed. Wells were washed with sterile PBS, fixed with methanol, stained with 0.1% crystal violet, rinsed, dried, and decolorized with 33% acetic acid. The stained biofilms were photographed, and the OD of each well was measured at 570 nm for statistical analysis.

### Fungal live/dead staining assay

To visualize drug-induced damage to fungal cells, a live/dead staining assay was performed on biofilms grown on coverslips. In a 24-well plate containing sterile coverslips, 1 mL of *A. fumigatus* (4 × 10⁶ CFU/mL) and 1 mL of each drug at 4× MIC were added to each well and incubated at 30 °C for 48 h. After removal of supernatants and gentle washing, biofilms on the coverslips were stained with DMAO and PI in the dark and incubated at room temperature for 20 min. The coverslips were then mounted on microscope slides, air-dried, and imaged under a CLSM. Both DMAO and PI are fluorescent nucleic acid dyes. DMAO stains both live and dead fungi green, whereas PI stains necrotic fungi red.

### Ultrastructural fungal morphology observation

Fungal ultrastructure after drug treatment was examined by electron microscopy. *A. fumigatus* cells (4 × 10⁶ CFU/mL) were incubated with antifungals (L-AmB, VCZ, RZF, or AFG) at 4× MIC for 48 h at 30 °C, harvested by centrifugation, and fixed in 2.5% glutaraldehyde. After fixation, samples were dehydrated through a graded ethanol series, critical-point dried, and platinum-sputtered. The treated specimens were examined using a field-emission SEM and a TEM.

### Macrophage cytokine mRNA measurement (qRT-PCR)

Based on the method of Li et al. with modifications^23^, RAW 264.7 cells (3 × 10^6^ cells/well) were seeded in 6-well plates and adhered overnight. Cells were then stimulated with *A. fumigatus* (2 × 10^7^ CFU/mL) and each drug at 40× MIC for 4 h at 37 °C. After stimulation, cells were harvested and total RNA was extracted using the RNAeasy™ Animal RNA Isolation Kit (Beyotime). One-step qRT-PCR was performed using a BeyoFast™ SYBR Green protocol on a LightCycler 96 thermocycler. The primer sequences for TNF-α, IL-1β, and the reference gene (GAPDH) are listed in Table 4^24^. Cycling conditions consisted of cDNA synthesis at 50 °C for 15 min, initial denaturation at 95 °C for 2 min, and 40 cycles of 95 °C for 15 s and 60 °C for 30 s. Relative mRNA expression levels were calculated by the 2^−ΔΔCt^ method.

**Table 4:** Primer sequences used for qRT-PCR analysis.

Gene	Sense primers (5ʹ-3ʹ)	Antisense primers (5ʹ-3ʹ)
IL-1β	ATCAGGACAGCCCAGGTCAA	GCCACCTTTTGACAGTGATGAG
TNF-α	ACCCTCACACTCACAAACCA	GCAGCCTTGTCCCTTGAAGA
GAPDH	GCATCTTCTTGTGCAGTGCC	GGTAACCAGGCGTCCGATAC

### Rabbit endophthalmitis model establishment

All animal experiments in this study were approved by the Animal Care and Use Committee of the Chinese Academy of Agricultural Sciences (IACUC of AMMS-11-2024-043). All methods were carried out in accordance with relevant guidelines and regulations. This study is reported in accordance with the ARRIVE guidelines. Twenty-five healthy adult male rabbits (1.4–1.6 kg) were purchased from Liaoning Changsheng Biotechnology Co., Ltd. (Liaoning, China), and were used to establish an *A. fumigatus* endophthalmitis model. Rabbits were anesthetized with isoflurane in oxygen delivered via a facemask (induction at 3–4% isoflurane and maintenance at 1.5–2.5% in 100% oxygen, oxygen flow rate 1.5 L/min). Under general anesthesia, each rabbit received an intravitreal injection of 100 µL *A. fumigatus* (3.9 × 10^4^ CFU/mL) in the right eye. The contralateral (left) eye did not receive any intravitreal injection. At 24 h post-infection (Day 0), the rabbits were randomly assigned to five groups (*n* = 5): Control (0.1 mL normal saline, NS), L-AmB (10 µg/0.1 mL), VCZ (50 µg/0.1 mL), RZF (35 µg/0.1 mL), and AFG (35 µg/0.1 mL). A single intravitreal injection was administered at this timepoint to assess therapeutic efficacy.

### Endophthalmitis clinical scoring

Following treatment on Day 0, the severity of intraocular inflammation was scored on Days 0, 1, 3, 5, and 7 according to the grading criteria in Table 5^10^. Scores at each time point were recorded and statistically analyzed.

**Table 5:** Clinical scoring scheme.

Grade	Cornea	Conjunctiva	Iris	Vitreous
0	Clear	Normal	Normal	Clear
1	Focal edema	Mild edema	Mild hyperemia	Areas of vitreous haze, some fundus details visible, good red reflex
2	Diffuse edema	Edema, mild hyperemia, slight exudate	Marked hyperemia	Moderate vitreous haze, no fundus details visible, partial red reflex
3	Opaque	Edema, marked hyperemia, heavy exudate	Marked hyperemia, synechiae, irregular pupil	No red reflex

### Aqueous humor cytokine measurement

On Day 7, under isoflurane anesthesia as described above, an anterior chamber paracentesis was performed on each infected eye to collect aqueous humor. Immediately after sample collection, the rabbits were euthanized by exposure to carbon dioxide in a gradually filled chamber until respiratory and cardiac arrest, in accordance with the AVMA Guidelines for the Euthanasia of Animals. The concentration of TNF-α in the aqueous humor of each eye was determined using a quantitative ELISA kit (Beyotime).

### Endophthalmitis antifungal efficacy evaluation

On Day 7, after CO_2_ euthanasia as described above, each infected eye was immediately enucleated and processed for fungal burden analysis following confirmation of the cessation of heartbeat and respiration as well as dilated and fixed pupils (to confirm complete death). Ocular tissues were aseptically minced and homogenized at 4 °C, serially diluted in NS, and spread onto PDA plates. After incubating inverted at 30 °C for 3 days, fungal colonies were counted. The fungal burden in each eye was expressed as CFUs, and the fungicidal rate was calculated as follows: Fungicidal Rate = (1 − CFUs of treatment group / CFUs of control group) × 100%.

### Histological analysis

On Day 7, after CO_2_ euthanasia as described above, the enucleated infected eyes were fixed. Ocular tissue sections were stained with H&E, PAS, and Masson’s trichrome to evaluate tissue morphology and the extent of fungal infiltration. Inflammation and damage were scored based on Table 6^25^ and statistically analyzed.

**Table 6:** Histopathological scoring scheme.

Score	Cornea	Conjunctiva	Iris/ciliary body	Vitreous	Retina
0	Normal	Normal	Normal	Normal	Normal
1	Focal thickening	Mild inflammation	Mild iritis	Occasional cells	Focal retinitis
2	Diffuse thickening	Moderate inflammation	Moderate iridocyclitis	Moderate cells or early fibrin membranes	Diffuse retinitis
3	Marked thickening	Marked inflammation	Severe iridocyclitis	Marked fibrinous membranes, cells	Marked necrosis

### Statistical analysis

The experimental data were analyzed using GraphPad Prism 8. One-way analysis of variance (ANOVA) was employed for multiple-group comparisons, and an unpaired Student’s *t*-test was used for two-group comparisons. Results are expressed as mean ± standard deviation (mean ± SD). *P < 0.05; **P < 0.01; ***P < 0.001.

## Data Availability

Data will be made available on request.
